# Strengthening post-graduate educational capacity for health policy and systems research and analysis: the strategy of the Consortium for Health Policy and Systems Analysis in Africa

**DOI:** 10.1186/s12961-016-0097-0

**Published:** 2016-04-12

**Authors:** Ermin Erasmus, Uta Lehmann, Irene Akua Agyepong, John Alwar, Don de Savigny, Peter Kamuzora, Tolib Mirzoev, Nonhlanhla Nxumalo, Göran Tomson, Benjamin Uzochukwu, Lucy Gilson

**Affiliations:** Health Policy and Systems Division, School of Public Health and Family Medicine, University of Cape Town, Cape Town, South Africa; School of Public Health, Faculty of Community and Health, University of the Western Cape, Cape Town, South Africa; Ghana Health Service/Department of Health Policy, Planning and Management, School of Public Health, University of Ghana, Legon, Ghana; Tropical Institute of Community Health and Development, Faculty of Health Sciences, Great Lakes University of Kisumu, Kisumu, Kenya; Health Systems Research and Dynamical Modeling Unit, Department of Public Health and Epidemiology, Swiss Tropical and Public Health Institute, Basel, Switzerland; Institute of Development Studies, University of Dar es Salaam, Dar es Salaam, Tanzania; Nuffield Centre for International Health and Development, Leeds Institute of Health Sciences, University of Leeds, Leeds, UK; Centre for Health Policy/MRC Health Policy Research Group, School of Public Health, Faculty of Health Sciences, University of the Witwatersrand, Johannesburg, South Africa; Health Systems and Policy Research Group, Department of Public Health Sciences, Karolinska Institutet, Stockholm, Sweden; Medical Management Centre, Department of Learning, Informatics, Management and Ethics, Karolinska Institutet, Stockholm, Sweden; Health Policy Research Group and the Department of Health Administration and Management, College of Medicine, University of Nigeria Enugu-Campus, Enugu, Nigeria; Department of Global Health and Development, Faculty of Public Health and Policy, London School of Hygiene and Tropical Medicine, London, UK

**Keywords:** Capacity development, CHEPSAA, Course review, Health policy and systems research and analysis, Low- and middle-income countries, Post-graduate, Teaching

## Abstract

**Background:**

The last 5–10 years have seen significant international momentum build around the field of health policy and systems research and analysis (HPSR + A). Strengthening post-graduate teaching is seen as central to the further development of this field in low- and middle-income countries. However, thus far, there has been little reflection on and documentation of what is taught in this field, how teaching is carried out, educators’ challenges and what future teaching might look like.

**Methods:**

Contributing to such reflection and documentation, this paper reports on a situation analysis and inventory of HPSR + A post-graduate teaching conducted among the 11 African and European partners of the Consortium for Health Policy and Systems Analysis in Africa (CHEPSAA), a capacity development collaboration. A first questionnaire completed by the partners collected information on organisational teaching contexts, while a second collected information on 104 individual courses (more in-depth information was subsequently collected on 17 of the courses). The questionnaires yielded a mix of qualitative and quantitative data, which were analysed through counts, cross-tabulations, and the inductive grouping of material into themes. In addition, this paper draws information from internal reports on CHEPSAA’s activities, as well as its external evaluation.

**Results:**

The analysis highlighted the fluid boundaries of HPSR + A and the range and variability of the courses addressing the field, the important, though not exclusive, role of schools of public health in teaching relevant material, large variations in the time investments required to complete courses, the diversity of student target audiences, the limited availability of distance and non-classroom learning activities, and the continued importance of old-fashioned teaching styles and activities.

**Conclusions:**

This paper argues that in order to improve post-graduate teaching and continue to build the field of HPSR + A, key questions need to be addressed around educational practice issues such as the time allocated for HPSR + A courses, teaching activities, and assessments, whether HPSR + A should be taught as a cross-cutting theme in post-graduate degrees or an area of specialisation, and the organisation of teaching given the multi-disciplinary nature of the field. It ends by describing some of CHEPSAA’s key post-graduate teaching development activities and how these activities have addressed the key questions.

## Background

The last 5–10 years have seen significant international momentum build around the field of health policy and systems research and analysis (HPSR + A) [1, 2], including greater consensus on definition and boundaries, the formation of Health Systems Global and three global symposia for sharing experiences in the field. Health Systems Global is a worldwide membership organization that brings together researchers, policymakers and implementers to promote health systems research and knowledge translation [3]. These developments complement the work of the Alliance for Health Policy and Systems Research since its inception almost two decades ago. The Alliance for Health Policy and Systems Research is a collaboration hosted by WHO and promotes health policy and systems research as a way to improve low- and middle-income countries’ (LMICs) health systems [4].

Over time, a core concern has been the need for capacity development in LMICs, comprising a focus not only on “*training competent cohorts of health systems analysts and researchers*”, but also on “*developing supportive and sustainable institutional settings and careers for research*” ([5], p. 5). In addressing LMIC capacity development needs, the importance of curriculum development for more substantive post-graduate HPSR + A training programmes rooted in social science perspectives, as opposed to short course training, has been specifically noted [6].

Social science perspectives are particularly important in understanding health policies and systems because they are socially constructed phenomena [7]. Moreover, it is through more substantive teaching programmes that the key concepts and boundaries of a knowledge and practice field are outlined. For HPSR + A, such teaching will “*promote a greater degree of shared perspectives, methodological understandings, and language among those who work in the field*” ([6], p. 4). Teaching will, in other words, give future researchers, educators, health system managers and health policymakers, a shared knowledge, language and understanding about how health systems work and health policies change, as well as approaches to researching them. Acquiring such knowledge is key to the emergence of future leaders in the field and, at the same time, good teaching will build greater demand for this field’s knowledge.

Post-graduate teaching is especially important for HPSR + A because the field draws on and brings together people from different disciplinary backgrounds and health system experiences, all of whom must develop the interdisciplinary understanding and skills that will enrich and deepen the knowledge and practice base [6]. The (real or virtual) classroom is an important space within which to connect researchers, managers and policymakers to cultivate the shared understanding and relationships that might culminate in policy-relevant research and the use of research in shaping policy formulation and implementation. Teaching is therefore important for its informative (acquiring knowledge and skills), formative (socialising participants into a community of professionals) and transformative (developing leadership and creating change) functions [8].

However, despite the importance of training and teaching, with a very few exceptions [9–11], there has so far been little collective reflection on and documentation of what is taught in this field, how teaching is carried out, the challenges experienced by educators and the future of HPSR + A teaching.

**Fig. 1:**
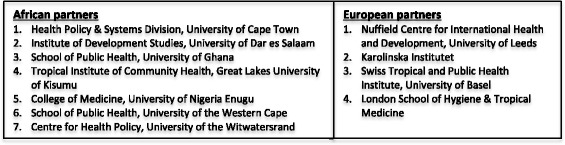
The CHEPSAA partners

For these reasons, the Consortium for Health Policy and Systems Analysis in Africa (CHEPSAA) made post-graduate curricula development and teaching a central focus of its work. CHEPSAA (2011–2015) was a consortium of seven African and four European universities that sought to extend sustainable African capacity to produce and use high-quality HPSR + A. Through various past projects with different organisational mixes, the African partners had a long history of working together on HPSR + A research and teaching and strong mutual knowledge and trust. The European partners were like-minded organisations, also with long histories of HPSR + A teaching and research, drawn from the African partners’ networks and able to work in the same collaborative, trust-based traditions. The consortium and its partners are described more fully elsewhere [2] (Fig. 1).

Indeed, CHEPSAA determined that “*strengthening the capacity of African universities is arguably a more sustainable strategy for developing the field of HPSR + A in Africa, than relying on training in high-income countries, and may also address the challenge of individually contracted research consultancies*” ([2], p. 832). Universities are mandated to teach the next generation of knowledge users and producers, as well as being involved in knowledge production.

This paper begins by outlining the methods and selected key findings of a situation analysis and course inventory of the post-graduate (primarily master’s-level) HPSR + A teaching of the CHEPSAA partner organisations. The information collected through this analysis and inventory formed the basis for planning and executing much of CHEPSAA’s teaching and curriculum development work and fed into a range of further products and processes. The closing sections of this paper discuss how these activities respond to and reflect what we know from the wider literature and CHEPSAA’s own work.

Drawing on these CHEPSAA experiences, the aim of the present paper is to support HPSR + A field-building by stimulating thinking about gaps in course content, prompting reflection on teaching practice, and generating ideas about how to improve and sustain HPSR + A post-graduate teaching. This paper is relevant to emerging and experienced HPSR + A educators, other faculty and university managers who influence how post-graduate courses are structured and implemented, and funders of HPSR + A capacity development initiatives.

The paper’s focus on HPSR + A stands in contrast to most of the available literature addressing capacity development for health in Africa [12–15], which focuses primarily on broader capacity development initiatives in fields such as population-based field epidemiology, demography and population studies, biostatistics, and public health. At the same time, the paper complements similar findings from an organisational capacity assessment [10] conducted by seven schools of public health in east and central Africa by covering different organisations and additional countries, as well as by considering specific course-level characteristics. This specific course-level focus also represents a level of assessment that complements the broader review of health policy and systems research training in LMICs conducted by the Teaching and Learning Health Policy and Systems Research Thematic Working Group of Health Systems Global [11]. This focus on HPSR + A teaching is, finally, also different from some of the most recent work on supporting the conduct and use of health policy and systems research [16], which has focused on understanding the topic areas (linked to the health system building blocks framework) on which academic and research organisations work and developing priority areas for future research among a range of health system stakeholders across different countries.

## Methods

CHEPSAA’s first collective activity in 2011/2 was to conduct HPSR + A capacity assets and needs assessments in the seven African member universities, the full details of which are reported elsewhere [2]. These assessments were the foundation that informed the design of the rest of CHEPSAA’s activities over the years. Using their findings, CHEPSAA implemented participatory and consultative processes to develop consortium-wide and partner-specific activities in relation to staff and organisational development, teaching capacity and curriculum development, and capacity for networking and getting research into policy and practice – areas through which CHEPSAA believed partners could be developed and the wider field of HPSR + A strengthened.

The assessments investigated six sub-themes, including the scope and quality of HPSR + A teaching. It was found that all the CHEPSAA members taught HPSR + A at post-graduate level, typically modules situated in wider degree programmes. The South African universities had better teaching infrastructure such as space, equipment and software. In most organisations, university funded staff taught HPSR + A, but in certain organisations it was necessary to use research grants to cross-subsidise teaching. It was clear from these assessments that partners’ existing educators and courses were assets, as was the demand for HPSR + A in all the African member countries [2]. Strategically, therefore, it made sense to capitalise on these assets to take forward work on HPSR + A teaching within CHEPSAA, which was the only teaching and capacity building network to which partners belonged.

The CHEPSAA team responsible for its work on teaching and curriculum development then collected additional information from partners that focused on their HPSR + A courses, but also included other aspects of teaching contexts and practices. The specific methods and significance of the situation analysis and course inventory must, however, be understood in the context of CHEPSAA’s wider methods, activities and processes. Overall, they sought to understand what partners were teaching and to determine how to work collaboratively to improve courses and teaching practices for the benefit of the CHEPSAA partners and the HPSR + A community in general (hence the open access nature of the new courses that were developed). The situation analysis addressed the following key questions:

- What subjects/courses are CHEPSAA partners teaching that are relevant to HPSR + A?
- Who are the courses’ target audiences or participants?
- How is this teaching funded and structured, including the institutional locations of teaching, the place of the teaching in post-graduate programmes and the time devoted to courses?
- How is this teaching carried out, including delivery mode, class activities and assessment practices?
- Do the CHEPSAA partners intend to develop their HPSR + A teaching and, if so, what support might they need?

Relevant information was collected in a cross-sectional way through two largely open-ended questionnaires that were completed by the principal investigators of each of the CHEPSAA partners. The first questionnaire was aimed at each partner organisation as a whole, while the second collected information on selected specific courses. To encourage uniformity in submissions, the CHEPSAA curriculum development coordinators developed a background document containing, among other things, a definition of HPSR + A, using the definition of the Alliance for Health Policy and Systems Research.^a^

Partners submitted, in varying degrees of detail, information on 104 courses in post-graduate programmes (the full selection). Using the core concerns of the definition of HPSR + A to make judgements, these 104 courses were first categorised into two broad groups: those that focused most directly on the core concerns of HPSR + A and those that appeared relevant to HPSR + A, but that might in the first instance be categorised as part of other fields, mostly public health. The former group contained 34 courses that were then grouped into three themes following further analysis of the course titles and objectives. One theme, health policy analysis, was clearly a common area of teaching in existing CHEPSAA partner programmes. The two other themes, however, represented two areas (understanding health systems, and health policy and systems research and evaluation) that were less well developed in these programmes.

**Fig. 2:**
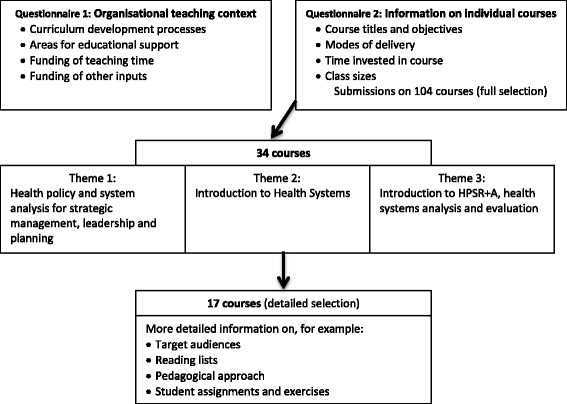
Collecting and analysing information on post-graduate teaching – process and focus

However, all three themes were identified by CHEPSAA as important to a future HPSR + A curriculum. Partners were then requested to submit more detailed information on courses within these themes as a basis for deciding how they could be further developed and strengthened through CHEPSAA collective work. However, as a number of the courses included in the first theme of health policy analysis were derivatives of an existing open access course previously developed by some of the partners, these courses were excluded from this second round analysis. Finally, submissions of varying degrees of detail were received with respect to 17 courses (the detailed selection). This process of information collection and analysis is summarised in Fig. 2.

Both questionnaires collected some basic qualitative information (e.g. open-ended statements about challenges), which was analysed by the CHEPSAA coordinators by inductively grouping materials into themes. Information of a more quantitative nature was, meanwhile, analysed by tabulating it against pre-specified variables (e.g. the number of courses delivered in classrooms vs. the number of distance learning courses). All analyses were then checked by the principal investigators of the CHEPSAA partners to confirm their validity.

Clearly, the findings of the situation analysis and course inventory are derived from a self-selected sample of university-based groups teaching HPSR + A and their experience is not necessarily generalizable to or replicable in other contexts. However, within their respective countries, the CHEPSAA partners are central to the teaching of HPSR + A and so their experiences, read in conjunction with similar literature, are valuable when considering stimulating further discussion of how to strengthen post-graduate teaching in the nascent field of HPSR + A.

Finally, as discussed in detail further on, this situation analysis and course inventory fed directly into a number of the other strands of CHEPSAA’s teaching and curriculum development work. First, it was the platform for developing two new open access master’s-level courses. Second, these courses were created in workshops in which senior and junior educators worked together to learn about the principles of curriculum development and then applied these principles to adapt existing course content and create new materials. The courses were then piloted, with the piloting explicitly including opportunities for junior educators to learn both subject knowledge and to develop teaching skills through apprenticeship. These curriculum development processes included participation from both African and European partners, and were led as a collective process. Following the consortium’s agreed principles, the primary role of the European partners was to support, not direct, these processes; therefore, they specifically helped to develop and pilot course materials and gave guidance on issues in which they had particular expertise. The course materials have also been subsequently incorporated into both the African and European partners’ teaching programmes, demonstrating their collective value. Finally, the situation analysis and course inventory informed a range of teaching-related documents developed by CHEPSAA to stimulate wider thinking, including concept notes on masters- and PhD-level training in HPSR + A.

## Results

This section begins by reflecting on the types of organisations in which HPSR + A post-graduate teaching is offered, the types of qualifications linked to the teaching and the students these courses are aimed at. It then moves to consider specific details of course content, delivery mode, time structures, teaching activities and assessments, before concluding with challenges and areas for further support.

### Organisations and types of qualifications

Most of the full selection of 104 courses (n = 58) were offered as part of Master in Public Health (MPH) degree programmes, reflecting the fact that CHEPSAA partners were often based in, and had links to, schools of public health. Some courses were, however, taught as part of MA (n = 29) or MSc (n = 27) degree programmes – for example, from the Institute of Development Studies at the University of Dar es Salaam (primarily a development, not health-focused, organisation) and the School of Government at the University of the Western Cape, which offers HPSR + A-related courses on advanced public policy analysis and management and development policy, planning and management. The latter was not a CHEPSAA partner, but its courses were included because, acknowledging the multi-disciplinarity of HPSR + A, we wanted to consider relevant teaching offered by other departments in CHEPSAA partner universities.

Just fewer than half of the courses (n = 49) were available as part of post-graduate diplomas or certificates offered by the universities of Leeds, Nigeria, the Western Cape and Cape Town. The total number of courses mentioned in this section exceeds the 104 unique courses identified in the work because, in some cases, most notably the University of Leeds, the same courses are offered as part of degree, diploma and certificate programmes.

### Students and target audiences

Qualitative analysis showed that the CHEPSAA partners often described the target audiences for their courses and the students who attend them through general terms such as public health practitioners and health professionals, although in some instances there were more specific descriptions involving functions or focal areas, for example, health policymakers, health managers, hospital and health facility managers, and managers of health programmes. The responses also included various references to courses being attended by existing or future researchers or educators.

Beyond this core, the target audiences were also sometimes described in terms of whether students were employed or not, the extent of their work experience, the country settings where students gained work experience, the economic sectors in which they worked or were being prepared to work, and their primary academic disciplines.

Reflecting diverse target audiences, educators therefore describe their students and the target audiences for their courses in different ways, using a variety of labels and constructs.

### Overall characterization of courses

Among the full selection of courses, there are two main dividing lines. First, there is a distinction between courses that focus on research methods, monitoring and evaluation, and those that focus on other subject content. A second dividing line is the extent to which courses mostly focus on the concerns of HPSR + A or whether these concerns occupy a more marginal place within them. The latter would include courses that, based on qualitative and interpretive judgements, do not directly or obviously focus on HPSR + A (e.g. qualitative research methods in general rather than a specific focus on HPSR + A research designs and methods) and those that seem to present their topics mostly from the perspective of another discipline (e.g. public health), rather than through the unique lenses of HPSR + A.

Ultimately, therefore, four groups of courses were identified:

1. HPSR + A subject courses that clearly address central topics of health policy and systems development (e.g. University of the Western Cape, Understanding and Analysing Health Policy);
2. HPSR + A subject courses where the focus is more on broader public health or development topics (e.g. University of Leeds, Health Promotion);
3. Research-related courses that are strongly focused on HPSR + A concerns (e.g. University of Cape Town, Introduction to Health Systems Research and Evaluation);
4. Research-related courses where HPSR + A concerns are more marginal (e.g. Great Lakes University, Advanced Research Methods).

### Delivery mode and class size

Despite the growing interest in the use of new teaching technologies, most of the courses in the full selection were delivered face-to-face in classroom settings. While there is nothing wrong with face-to-face teaching per se, this might indicate that educators are not keeping up with new technological developments or using the full range of tools at their disposal. A smaller group of courses were offered in blended learning mode, involving both classroom settings and distance learning and, finally, a few courses were offered only through distance learning. All the distance learning courses were provided by the University of the Western Cape, reflecting this organisation’s unique approach and contribution within CHEPSAA.

It is clear that class sizes change from year to year. However, by far, the most typical class size is 20–30 students per course per year. Also fairly typical is a second set of courses with 10–20 students. Few courses drop below 10 students or have more than 30 students (although the highest reported class size was 51).

### Time structures of courses

Among the full selection of courses, there was considerable diversity in the amount of time allocated to HPSR + A-relevant courses and in how that time is organised, both within and between universities. Nonetheless, the data revealed three broad groups.

First, the courses most typically provided 30–40 hours of contact time between educators and students. The total overall notional time commitment of these most typical courses (the total time students are expected to spend on all aspects of the course), meanwhile, fell between 112 and 200 hours, indicating that the majority of time in any course is self-study time.

This balance between contact time and self-study tasks, however, varies considerably even within the same organisation. For example, one CHEPSAA partner offers courses with total notional time commitments of 150–200 hours, within which the contact hours can vary from 10 to 50 and the self-study time from 30 to 100 hours. Furthermore, another partner offers courses with total notional time commitments of 150 hours, within which the contact time can range from 30 to 60 hours.

Second, a small number of courses (n = 5) had 165 hours of contact time, nested in a notional time commitment of 300 hours.

A third, small (n = 3) group had lecturing and group work of 72–83 hours, but also with a 300-hour total notional time commitment.

### Teaching activities and assessments

Information on teaching approaches is drawn from the detailed selection of courses. The majority of teaching activities identified comprised lectures, group activities and discussions, and seminars. A number of the lectures were qualified as being ‘interactive’, while it was also clear that at least in some of the seminars students were expected to play an active part in leading and contributing to the discussions. Some of the less typical activities mentioned were roleplay and the use of student diaries for reflection on topics raised in the course.

While almost all the activities seemed classroom-based, there were a few mentions of site visits and internet-based learning and interaction. A small number of submissions also mentioned the use of media such as videos, in addition to perhaps more traditional media such as textbooks and journal articles. We did not collect information to fully account for what students were expected to do during the large portion of self-study time and whether these activities were well integrated with the teaching activities undertaken during contact hours; however, completing course assignments was a common activity across courses.

Individually written essays or reports were noted as the predominant form of assignment (used in almost all of the detailed selection of 17 courses). About a third of the courses (n = 5) used group presentations (one used a group report). A smaller number (n = 4) of courses also used shorter pieces of written work such as reflective logs or notes, the task of formulating research questions, and brief reflections on journal articles.

Across the essay/report-type assessments, students have a lot of freedom to choose the topics they will address, for example, by choosing the country on which to write a case study or being allowed to choose any substantive topic or problem of relevance to the course and assignment (sometimes with the guidance/approval of an educator). Sometimes students are given specific real or hypothetical scenarios as background, while still having substantial scope for choosing which aspects of the scenario to address. Students are always given guidance on how to structure the assignments and what to include in them, but there is variation in the level of guidance (ranging from an outline of broad sub-headings to more detailed guidance on what might/should be included under each sub-heading).

In approximately half the courses in the detailed selection it was also clear that essay/report-type assignments were not isolated from the rest of the course. Students were, for example, able to write assignments on topics linked to larger dissertations or research projects, were able to link their individual assignments to group work tasks or were able to complete assignments in phases, e.g. submitting a piece of written work, getting feedback and then submitting a follow-up piece of work building on the first.

Only about a third of the courses in the detailed selection used examinations as a form of assessment. For those using exams, the exam weightings clustered around 40–50% of the total course mark, except for one course where the exam accounted for 100% of the course mark.

Finally, there was diversity in the frequency of assessment. Similar numbers of courses (3–4) had between one and three assessments. Two courses had four assessments and one had no assessment, with the idea that students would use the knowledge gained in this research methodology course to improve their dissertations. Research methodology courses tended to have fewer assessments than other courses (one had no assessment and about half of the courses in this category had 1–2 assessments), and often the assessment task was to produce a protocol, plan or strategy for research, monitoring or evaluation work.

### Challenges and issues for further support

Partners also reflected on key areas in which they would require support to develop their HPSR + A teaching, with qualitative analysis revealing a number of themes.

In general, the European partners reported fewer challenges and areas for support. The most notable theme was funding, which was mentioned in relation to securing funding for students from abroad and the funding models of courses, with the need to find sponsors as not all courses received government funding.

Among the African partners, funding also emerged as a first key challenge. Some partners’ teaching time was funded through government or their employing organisations, while others reported that it was necessary to use research grants and consultancies to cross-subsidise curriculum development, teaching and student supervision (see also [2]). With respect to short courses, only about half the principal investigators indicated that they always or usually fully recover their running costs. When organisations do not fully recover their teaching costs or cross-subsidise them through research funding, which can be unpredictable, this can undermine the financial viability of groups or limit the extent to which teaching is institutionalised in wider university structures, constraining the capacity to continue or expand teaching.

Second, African partners noted their concerns about, and that they would value further support in, curriculum and course material development. In the context of limited staff, limited funding and multiple time commitments, people were unsure of how to proceed to develop a wider suite of HPSR + A courses, what the most efficient and effective ways were to access curricula and course materials, and whether it would be possible and wise to import and adapt existing materials from other contexts, how to carve out more time for the time-consuming tasks of curriculum and materials development, and how to go about developing and strengthening organisational processes for regularly reviewing and updating curriculum content.

Additional, less prominent concerns among the African partners were improving the capacity and expertise of those who teach in this field (both senior staff who are interested in HPSR + A, but whose training and experience might have been grounded in another discipline, and younger educators who need more subject knowledge and teaching experience), developing or improving quality assurance processes around curriculum development and teaching, improving student supervision, and developing alternative ways of assessing student performance.

## Discussion

The CHEPSAA findings, together with the other two key, related pieces of work [10, 11], identify the following six characteristics, and challenges, of post-graduate HPSR + A education in Africa:

- The range and variability of courses addressing HPSR + A and variation in the extent of focus on HPSR + A in existing courses, reflecting the still emerging boundaries of this field;
- The important, but not exclusive role of schools of public health in offering HPSR + A post-graduate training;
- The variation across universities in the credit hours required to complete an MPH degree [10] and in the credit hours of specific HPSR + A courses, even those addressing the same subject area;
- The diversity of target audiences and variability in potential demand for HPSR + A courses;
- The limited availability of distance and non-classroom learning activities, although the majority of teaching time occurs outside the classroom in all courses and despite the growing importance of new teaching technologies; and
- The predominance of rather old-fashioned teaching styles, in the form of lectures and group discussions as teaching activities, and of assessment styles (including the focus on written assignments in the form of essays and reports and, in some programmes/courses, a fairly strong weighting on written exams [10]).

Looking ahead, therefore, these existing analyses of HPSR + A teaching programmes and capacity, as well as an analysis of teaching about LMIC health systems in the context of Australian public health academic programmes [9], suggest that three sets of key questions must be addressed in any effort to support African and other educators in further developing their HPSR + A post-graduate teaching. Such efforts must also be accompanied by reflection on the staff, funding and other resource challenges educators face, and attempts to overcome these where relevant [2, 10].

The three questions are:

- How can HPSR + A curriculum development address key aspects of current educational practice?
- Is it better to teach HPSR + A as a cross-cutting theme within a master’s programme, through focused courses or a combination of both?
- Do schools of public health and similar organisations have enough staff from different disciplines to offer the best possible teaching on health systems, and what challenges are faced in trying to work across disciplines and departments?

Table 1 outlines the origins and significance of these questions.

**Table 1:** Key questions to address in supporting the development of post-graduate HPSR+A teaching

Question 1: How can HPSR+A curriculum development address key aspects of current educational practice?	This question is central to consideration of what is currently being taught under the label of HPSR+A , how this teaching is done and how it might look in future. CHEPSAA’s analysis suggests that such curriculum development needs to address issues such as the diverse student groups of HPSR+A courses, the variation in credit hours for HPSR+A subject matter, limited student-educator contact time and the large portions of time allocated to other tasks, and forms of teaching and assessment. The question encourages consideration of how these issues should be dealt with, what current practices should be carried over to the future, and how current approaches can be optimised and new ones encouraged.
Question 2: Is it better to teach HPSR+A as a cross-cutting theme within a master’s programme, through focused courses or a combination of both?	CHEPSAA’s analysis shows that much HPSR+A teaching takes the form of courses that are situated in larger programmes such as MPH degrees, that they address diverse student audiences and that there is a large variety of courses with various degrees of HPSR+A focus. It is important, therefore, to think about the structures within which those courses fit. A key question in this regard is whether the field and its target audiences are best served through cross-cutting or more specialist courses. Teaching in a cross-cutting way will, for example, expose a wider range of students to the subject, while focused courses offer greater depth.
Question 3: Do schools of public health and similar organisations have enough staff from different disciplines to offer the best possible teaching on health systems, and what challenges are faced in trying to work across disciplines and departments?	As is clear from the definition used in this work, HPSR+A defines itself as a multi-disciplinary field. It has also been shown that researchers and educators in the field often want to increase multi-disciplinary work, but face challenges in seeking to do this, including having too little time for the course materials they aim to cover without even bringing in materials and perspectives from different disciplines and limited cross-disciplinary connections within their institutions or links with potential collaborators from other disciplines [11]. Given HPSR+A’s commitment to multi-disciplinarity, it is important to consider how this principle is addressed and brought to life in current and future teaching.

### Tackling the challenges: CHEPSAA’s response

CHEPSAA’s work on curriculum development and capacity building for HPSR + A teaching touched on all the above questions. In summary, and to briefly restate, CHEPSAA developed and published two masters-level courses, entitled Introduction to Complex Health Systems and Introduction to Health Policy and Systems Research, through a participatory process involving CHEPSAA partner staff in materials’ development and pilot testing. These newly developed courses complement Health Policy and Policy Analysis, a masters-level module published earlier. All are available as open access materials, under a Creative Commons licence, with facilitators’ notes, from CHEPSAA’s website, along with various other documents relevant to HPSR + A teaching capacity outlined below.

These courses specifically address critical gaps in the current suite of HPSR + A courses being offered by CHEPSAA partners, and together lay a foundation, including for designing related research, that draws on social science perspectives for understanding and analysing health policy and systems. Assuming no prior knowledge of their subject area, all can be taken by students from diverse backgrounds; but as a set, taken together, they can be seen as the core of a specialist HPSR + A masters programme. They would be well complemented by courses on specific health systems areas or issues as well as more specific research methods courses. CHEPSAA did not, therefore, take a deliberate stand on whether to teach HPSR + A as a cross-cutting or specialist area, but generated courses that could be used in either way. It did, however, consider the advantages, disadvantages and possible options for developing a specialist masters in the field, and developed a short briefing note on this issue [17]; some CHEPSAA partners have also begun discussion on what a professional doctorate in the broad area of health policy and systems might look like [18]. In addition, CHEPSAA generated a list of HPSR + A competencies that could guide further development of specialist programmes, as well as offer guidance for the development of other specific courses [19].

The participatory process applied in developing the course materials, meanwhile, began to address the concern that teaching staff lack the skills needed to teach multi- or inter-disciplinarity in HPSR + A and lack experience in curriculum development. One indicator of the value of these workshops is that in several CHEPSAA partners ‘step-down’ curriculum development workshops were run with wider groups of staff to share learning and broaden exposure to the curriculum development principles [20].

All three open access courses are designed around a total of 150 notional hours, including 30 hours of contact time; but they have all also been run as 4.5-day short courses (without assessments). CHEPSAA colleagues agreed that longer courses would provide a stronger introduction to the subject matter of focus, and would signal the need for, and encourage the allocation of, greater time to HPSR + A teaching across universities. In their design, the courses, thus, signal a new approach to HPSR + A training.

Indeed, in developing these new courses, CHEPSAA confronted many of the issues and challenges highlighted by the course inventory. The core issues addressed during the curriculum development workshops, and the materials used, formed the basis of a short guidance document on the principles and practice of good curriculum design for HPSR + A [21]. We recognised, for example, the diverse target audiences that would take these courses, and the particular challenges likely to be faced by students from a more bio-medical background in understanding social science concepts and working with discursive texts.

Course learning outcomes, therefore, address different levels of understanding as well as combining knowledge and practice outcomes, and we identified core threshold concepts for each course to signal the critical learning points of each (Table 2). Threshold concepts are foundational ideas that irreversibly transform students’ understandings of the subject and the world [22]. Course design is framed around these central points and outcomes, and supports the scaffolding of learning by students through iterative engagement with materials, concepts and practice approaches, and iterative assessment of knowledge and skills development. Scaffolding is about building on what students know already to support them in learning something new. It prompts the educator to think carefully, among other things, about how course content, exercises and tasks are sequenced [23, 24].

**Table 2:** Examples of threshold concepts from CHEPSAA’s courses

Threshold concepts shared across the courses	
• Health policies and systems are socially constructed; they exist within contexts and histories and are driven by and impact on a range of agents • Health systems comprise interacting dimensions of ‘hardware’ and ‘software’ • People are at the centre of the health system, driven by values and contexts • The health system is knowable and changeable	
Selected threshold concepts unique to the courses	
Introduction to Complex Health Systems	Introduction to Health Policy and Systems Research
Health systems are integrative by nature, and consist of complex inter-relationships; we all have a role in the system	HPSR + A is intentionally multidisciplinary and embraces multiple perspectives
Health system effectiveness is a ‘whole system’ judgement rather than one based on the effectiveness of specific interventions	Health care services/interventions/programmes provide a lens through which to investigate policy and systems issues (i.e. they are not the primary focus of the research)
People make sense of the system around them and act based on their understandings and mind sets	Good (i.e. sound) research design requires that the study design fits the question, issues and purpose
Power is everywhere: in agency, service delivery and decision-making	There is no hierarchy of study design in terms of quality and rigour in HPSR + A; and quality and rigour are always important
Everyone has a part to play in the system, working towards shared goals	Researcher curiosity, attentiveness and reflexivity are the basis of rigorous HPSR + A
The health system is a complex adaptive system	Theoretical ideas and concepts have value (as a guide for study design and analysis in HPSR + A)

The teaching approach proposed for contact time is deliberately participatory, seeking to build on existing, variable student knowledge, and also allow the sharing of experience that deepens understanding. Lectures are, therefore, combined with a range of individual and small group exercises that allow for deeper learning and the development of relevant practice skills (such as stakeholder analysis or developing substantively relevant research questions), either introducing or wrapping up topics and sessions.

The course materials and detailed facilitators’ guides also provide a range of suggestions of ways of bringing life to lectures (such as the use of video material) and ideas for how to use the self-study time to build on or prepare for classroom activities, including assessments that support scaffolded learning, to encourage active learning across the total notional hours allocated to each course.

In each course a core group work project complements other group tasks and provides a critical opportunity for authentic learning, by presenting real-world cases for analysis in small groups which also provide the basis for students’ personal thinking about, for example, how to analyse situations, manage differently or develop their own research protocol. A key pillar of authentic learning is for students to apply their knowledge to real-world problems and to undertake activities that are actually used in practice in their own fields and contexts [21]. The topics of the real-world cases include the Tanzanian Community Health Fund and lay boards, the additional duty hours allowance in Ghana, health facility committees and financial management in Kenya, and the implementation of the Patients’ Rights Charter in South Africa.

Beyond course design, CHEPSAA’s work reaffirmed a central finding of the broader health capacity development literature [6, 10, 12, 14, 25]: that is, the need for sustained funding. This was identified as a key risk for some CHEPSAA partners as many HPSR + A units within universities have historically been entirely or almost entirely grant-funded, receiving little core funding from their broader organisations and, in practice, cross-subsidising their teaching function through research grant income [2]. Funding is essential in creating the necessary organisational infrastructure and facilities for improved teaching, establishing the necessary posts, recruiting high-quality staff, developing staff skills, and expanding teaching. One CHEPSAA partner offers some hope in this regard, since, through designing a careful business case showing the number of hours each grant-funded researcher in the organisation spent on teaching, which quantified to full posts, it was able to secure two university funded teaching posts for the future. Sharing this sort of experience as well as advocating for increased domestic and international funding for all work related to HPSR + A remains a vital strategy in building the field [26].

## Conclusions

The CHEPSAA experiences reported in this paper add insights to existing literature about the current situation of post-graduate HPSR + A education in Africa and LMICs more generally. They also offer ideas about how to strengthen these educational activities – both in the form of the open access materials available and in the processes through which CHEPSAA developed these materials and so exposed future African educators to critical principles of curriculum design and teaching practice. The courses produced are, of course, only the first wave of newly designed HPSR + A educational materials and will be further developed and strengthened by others as they use them. A stronger and dedicated effort is also needed to develop the skills and practices of African HPSR + A educators through formal training and through further peer networking. However, the CHEPSAA courses or parts of them have already been introduced into the teaching programmes of almost all the CHEPSAA partners in both Africa and Europe and thus far the courses Introduction to Complex Health Systems and Introduction to Health Policy and Systems Research have been downloaded 277 and 217 times, respectively, from 55 countries. The ideas embedded in these courses, showing how social science perspectives offer value to HPSR + A as well as how to structure related teaching, will have a life of their own, shaping and influencing wider thinking and teaching practice. We encourage comment and reflection on experience of their use on our website [27] or via the CHEPSAA twitter handle [28].

## Endnote

^a^ “*Health policy and systems research (HPSR) is an emerging field that seeks to understand and improve how societies organize themselves in achieving collective health goals, and how different actors interact in the policy and implementation processes to contribute to policy outcomes. By nature, it is inter-disciplinary, a blend of economics, sociology, anthropology, political science, public health and epidemiology that together draw a comprehensive picture of how health systems respond and adapt to health policies, and how health policies can shape − and be shaped by − health systems and the broader determinants of health. Health policy and systems research can be employed at several points in the policy cycle, from getting an issue onto the policy agenda to evaluating and learning from implemented policies. In this way, HPSR is characterized not by any particular methodology, but the types of questions it addresses. It focuses primarily upon the more upstream aspects of health, organizations and policies, rather than clinical or preventive services or basic scientific research (for example into cell or molecular structures). It covers a wide range of questions − from financing to governance − and issues surrounding implementation of services and delivery of care in both the public and private sectors. It is a crucial policy analysis tool − of both policies and processes − including the role, interests and values of key actors at local, national and global levels. The appropriate mix of disciplines to be used in HPSR depends largely on the nature of the research question being addressed…*” [29].
